# Long-term endocrine and reproductive outcomes in female adolescent and young adult survivors after hematopoietic stem cell transplantation for hematological malignancies during childhood or adolescence

**DOI:** 10.3389/fendo.2026.1921689

**Published:** 2026-10-15

**Authors:** Weronika Zajączkowska, Katarzyna Derwich, Cezary Miedziarek, Agnieszka Sobkowiak-Sobierajska, Zofia Kolesińska, Maria Buda, Witold Kędzia, Karina Kapczuk

**Affiliations:** 1 Division of Gynecology, Poznan University of Medical Sciences, Poznan, Poland; 2 Doctoral School, Poznan University of Medical Sciences, Poznan, Poland; 3 Department of Pediatric Oncology, Hematology and Transplantology, Institute of Pediatrics, Poznan University of Medical Sciences, Poznan, Poland; 4 Division of Gynecological Oncology, Department of Gynecology, Poznan University of Medical Sciences, Poznan, Poland; 5 Department of Pediatric Endocrinology and Rheumatology, Poznan University of Medical Sciences, Poznan, Poland; 6 Division of Gynecology, Unit of Pediatric and Adolescent Gynecology, Poznan University of Medical Sciences, Poznan, Poland

**Keywords:** bone mineral density (BMD), childhood cancer survivors (CCS), fertility preservation and restoration for cancer patients, gonadotropin releasing hormone (GnRH) agonist, hematopoietic stem cell transplantation (HSCT), ovarian reserve (OR), premature ovarian insufficiency (POI), transient ovarian insufficiency (TOI)

## Abstract

**Background:**

Cancer treatment during childhood and adolescence, particularly hematopoietic stem cell transplantation (HSCT), may adversely affect long-term reproductive and endocrine health in female adolescent and young adult (AYA) survivors, leading to premature ovarian insufficiency (POI), which may be associated with impaired pubertal development, low bone mass, and reproductive complications.

**Methods:**

This study included 34 female survivors treated with HSCT for hematological malignancies during childhood or adolescence (median age 10.8 years). The median follow-up from HSCT to the last gynecological assessment was 8.5 years. Hormonal assessment, pelvic ultrasound, and bone mineral density (BMD) evaluation were performed. Patients were classified as having normal ovarian function, transient ovarian insufficiency (TOI), or persistent POI.

**Results:**

At the last follow-up, normal ovarian function was observed in 15 patients (44.1%), TOI in 7 (20.6%), and persistent POI in 12 (35.3%). Patients with normal ovarian function were significantly older at HSCT (p = 0.0432), whereas premenarchal status was associated with impaired ovarian outcome (p = 0.0066). In multivariable analysis including cyclophosphamide equivalent dose (CED), radiotherapy, and menarchal status at HSCT, postmenarchal status was associated with markedly lower odds of ovarian insufficiency (adjusted OR 0.056, 95% CI 0.005–0.566; p = 0.0147). All postmenarchal patients received GnRHa, precluding separation of the effects of menarchal status and GnRHa exposure; GnRHa-treated survivors had higher AMH concentrations (p =0.0032). AMH and FSH concentrations differed significantly between groups (both p < 0.01). Uterine and mean ovarian volumes, antral follicle count (AFC), and BMD were significantly lower in patients with TOI and POI (all p < 0.05). Radiotherapy exposure was associated with both lower uterine and ovarian volumes, while puberty induction was also associated with lower uterine volume (all p <0.05). Two patients diagnosed with POI achieved spontaneous pregnancies during follow-up, one resulting in a live birth.

**Conclusions:**

Ovarian dysfunction is highly prevalent after childhood HSCT, although partial recovery of ovarian function may occur even in patients with a diagnosis of POI. Persistent POI is associated with impaired uterine development and lower BMD despite ongoing hormone replacement therapy, underscoring the need for timely and long-lasting individualized endocrine and reproductive care in AYA survivors.

## Introduction

Hematopoietic stem cell transplantation (HSCT) has become a curative treatment for numerous hematologic malignancies and other severe pediatric disorders, resulting in a growing population of long-term adolescent and young adult (AYA) survivors. However, conditioning regimens required for successful engraftment, particularly high-dose alkylating chemotherapy and total body irradiation (TBI), are associated with substantial gonadotoxicity (1) and may adversely affect long-term reproductive and endocrine health (2, 3).

Premature ovarian insufficiency (POI) is among the most important late complications observed in female HSCT survivors. In addition to infertility, POI may lead to impaired pubertal development, amenorrhea, reduced bone mineral density, cardiovascular complications, and diminished quality of life (4). Furthermore, gonadotoxic treatment may also impair uterine development and disrupt the hypothalamic–pituitary–ovarian axis, thereby contributing to adverse reproductive outcomes (5).

The extent of gonadal damage depends on both treatment- and patient-related factors. Alkylating agents, particularly cyclophosphamide and busulfan, induce DNA damage and apoptosis in ovarian follicles, whereas TBI further increases the risk of ovarian failure because ovarian tissue is highly radiosensitive (6, 7). Although younger age at treatment has traditionally been considered relatively protective because of the larger primordial follicle pool in prepubertal girls, ovarian damage may still occur despite premenarchal status (8). Moreover, cumulative gonadotoxic exposure may be increased by chemotherapy administered before HSCT.

Despite increasing awareness of these complications, long-term ovarian outcomes after childhood HSCT for hematological malignancies remain insufficiently characterized. Studies in this population have reported POI rates of approximately 70–90% (9, 10); however, prevalence varies depending on conditioning regimens, follow-up duration, and definitions of ovarian dysfunction. Importantly, ovarian insufficiency after gonadotoxic treatment may not always be irreversible. Previous studies have described cases of transient ovarian insufficiency (TOI), characterized by partial recovery of ovarian function after an initial diagnosis of POI (8, 11). However, data regarding the long-term endocrine, reproductive, and ultrasound characteristics of these patients remain limited.

The aim of this study was to evaluate long-term endocrine and reproductive outcomes, including ovarian reserve, uterine development, and bone mineral density, in AYA survivors treated with HSCT for hematological malignancies during childhood or adolescence.

## Materials and methods

This was a retrospective–prospective observational cohort study. Patients who underwent HSCT for hematological malignancies at the Department of Pediatric Oncology, Hematology and Transplantology, Poznan University of Medical Sciences, were included. Long-term follow-up, including clinical, hormonal, and radiological assessments, was conducted in the Gynecological and Obstetric Clinical Hospital, Poznan University of Medical Sciences.

Patients were eligible for inclusion if they met all of the following criteria:

1. Female sex
2. History of HSCT for hematological malignancies between 2000 and 2024
3. Age 11–35 years at the study enrollment
4. ≥12 months since completion of all oncological treatments, including HSCT.

Exclusion criteria were as follows:

1. Current pregnancy or lactation;
2. History of bilateral gonadectomy;
3. Pre-existing genetic or medical conditions associated with an increased risk of POI (e.g., Turner syndrome, Fragile X syndrome, Fanconi anemia);
4. Use of combined oral contraceptives (COCs) within 3 months prior to evaluation.

Patients receiving hormone replacement therapy (HRT) for previously diagnosed POI were not required to discontinue treatment to maintain clinical stability and ensure adequate bone protection.

## Clinical and laboratory assessments

Clinical evaluation included a detailed medical history focusing on oncologic treatment, radiotherapy exposure, and the use of gonadotropin-releasing hormone agonists (GnRHa) during therapy, as well as on pubertal development and menstrual patterns.

Hormonal assessment included serum concentrations of follicle-stimulating hormone (FSH), estradiol (E2), and anti-Müllerian hormone (AMH) measured using electrochemiluminescence immunoassays (ECLIA). Due to irregular or absent menstrual cycles and limited patient availability during follow-up appointments, hormonal analyses were conducted regardless of cycle phase and on random days within the cycle.

Uterine and ovarian imaging was performed using a Voluson E8 ultrasound system (GE Healthcare, Zipf, Austria). A C1–6 convex transducer was used for transabdominal ultrasound, whereas a RIC5–9-D transducer was used for transvaginal ultrasound, depending on patient characteristics. Two investigators performed the examinations. Uterine length (L) (including length of the body and of the cervix), the anteroposterior (AP) and the transverse (T) diameters of the uterine body and of the cervix were measured. Uterine volume (UV) was calculated by assuming the form of the uterine body and of the cervix to be ellipsoid using the volume formula: L x AP x T x π/6 (0.523). The volumes of the uterine body and of the cervix were summed. Ovaries were measured in three perpendicular diameters (D1, D2, D3) and ovarian volumes were calculated using the ellipsoid formula: D1 x D2 x D3 x π/6 (0.523). Mean ovarian volume was subsequently calculated as the average of the volumes of both ovaries. The antral follicle count (AFC) was defined as the number of antral follicles (AF) measuring 2–9 mm in diameter visualized in each ovary. Total AFC was expressed as the sum of AF counted in both ovaries.

CED was calculated using a validated algorithm based on the Childhood Cancer Survivor Study methodology (12) and implemented in an online fertility risk calculator to quantify cumulative exposure to alkylating agents. CED captured the total exposure to alkylating chemotherapy administered throughout the entire course of oncological treatment, including both pre-transplant therapy and conditioning regimens. Risk of gonadotoxicity was classified using previously published stratification models, with age-specific thresholds based on menarchal status at the time of treatment. For postmenarchal patients, CED values of <4 g/m², 4–8 g/m², and >8 g/m² were considered low-, intermediate-, and high-risk, respectively. For premenarchal patients, the corresponding thresholds were <8 g/m², 8–12 g/m², and >12 g/m² (13).

For patients who underwent two HSCT procedures, age at HSCT was defined as the age at the first transplantation and was used in all analyses involving age at HSCT.

Bone mineral density of the lumbar spine (L1-L4) was assessed using dual-energy X-ray absorptiometry (DXA) with a Lunar Prodigy Primo densitometer, and results were reported as absolute values and Z-scores.

Only survivors aged 11–35 years at follow-up were included in the study. This lower age limit was selected in accordance with the International Late Effects of Childhood Cancer Guideline Harmonization Group (IGHG) recommendations, which advise surveillance of pubertal development in girls at risk of treatment-related POI from approximately 11 years of age, with hormonal evaluation in those who fail to initiate puberty (absence of Tanner stage B2 breast development) or fail to progress through puberty for ≥12 months (14).

POI was diagnosed according to the ESHRE criteria (4, 15). In postmenarchal patients, the diagnosis required amenorrhea lasting more than four months and FSH concentrations >25 IU/L, confirmed in two measurements obtained at least 4 weeks apart. In premenarchal girls, the diagnosis required hypergonadotropic hypogonadism, defined as two serum FSH measurements >25 IU/L obtained at least 6 weeks apart, low serum estradiol concentrations, and persistent absence of pubertal breast development (Tanner stage B1). All hormonal assessments used to establish the diagnosis of POI were performed before HRT. Pubertal induction was initiated only after these diagnostic criteria were met, in accordance with ESHRE recommendations for the management of girls with established POI (4). Chronological age alone was not used to establish the diagnosis of POI or to initiate pubertal induction.

TOI was defined as the partial recovery of ovarian function following a previous diagnosis of POI, characterized by spontaneous menstrual cycles, spontaneous pubertal progression, the presence of AF on ultrasound, and FSH levels below 25 IU/L without HRT.

This study was conducted and reported in accordance with the Strengthening the Reporting of Observational Studies in Epidemiology (STROBE) guidelines for observational studies (16).

The study was approved by the local Ethics Committee (approval number 840/23). Written informed consent was obtained from all participants or their legal guardians.

Statistical analysis was performed using RStudio with R version 4.2.2. Continuous variables were summarized as medians with interquartile ranges (IQRs), while categorical variables were presented as counts and percentages. Missing data were excluded; therefore, the number of observations differed between individual analyses. For comparisons of continuous variables across three groups, the Kruskal–Wallis test was used. When statistically significant differences were identified in the global test, *post-hoc* pairwise comparisons were performed using Dunn’s test with Benjamini–Hochberg correction for multiple comparisons. For comparisons between two independent groups, the Wilcoxon rank-sum test was applied, while paired comparisons between two time points were performed using the Wilcoxon signed-rank test. Categorical variables were compared using the chi-square test or Fisher’s exact test, depending on expected cell counts, with Fisher’s exact test used when expected frequencies were low.

Multivariable logistic regression was performed to assess factors independently associated with ovarian insufficiency, defined as TOI or POI versus normal ovarian function. CED, radiotherapy exposure, and menarchal status at HSCT were included as covariates. Adjusted odds ratios (ORs) with 95% confidence intervals (CIs) were calculated. Overall model significance was assessed using the likelihood-ratio test, and model discrimination was evaluated using the area under the receiver operating characteristic curve (AUC) with 95% CI.

The association between total AFC and mean ovarian volume was assessed using Spearman’s rank correlation and linear regression. All tests were two-sided, and p-values below 0.05 were considered statistically significant. Adjusted p-values were reported for *post-hoc* pairwise comparisons. Data visualization was performed using violin plots combined with boxplots and individual data points, with medians and group-specific sample sizes shown on the plots. The following R packages were used for data visualization: ggplot2, ggpubr, rstatix, and patchwork.

## Results

A retrospective review of the institutional database identified 192 female patients who underwent HSCT between 2000 and 2024. Of these, 109 met the inclusion criteria of a hematological malignancy and age 11–35 years at follow-up. Twenty-three patients had died before follow-up, leaving 86 eligible survivors. Thirty-five could not be contacted, and 17 declined to participate. The study was completed by 34 participants who provided written informed consent (**Figure 1**; **Tables 1**–**3**).

**Figure 1:**
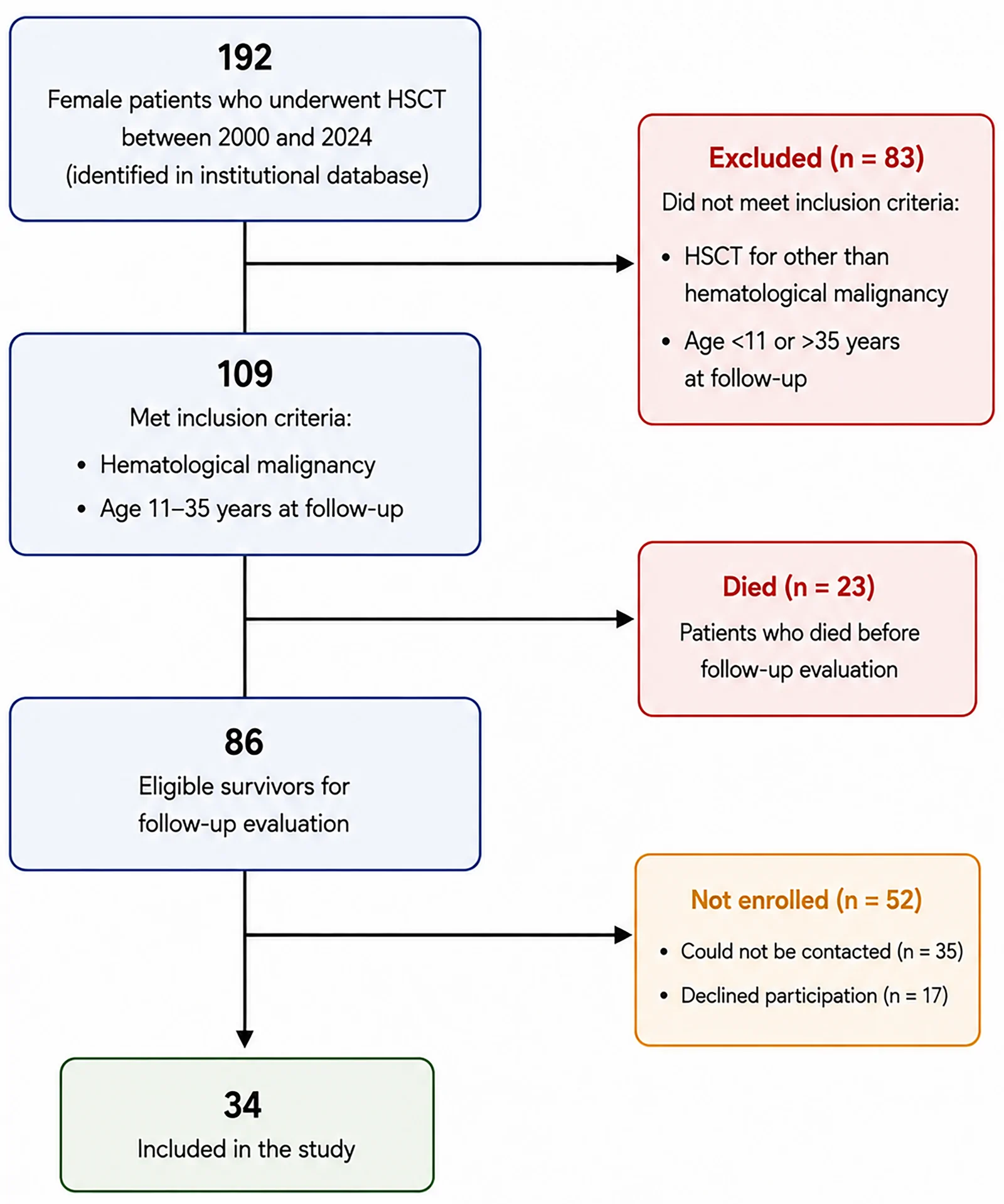
Flow of study participants. HSCT, hematopoietic stem cell transplantation.

**Table 1:** Characteristics of 34 patients.

No	Age at diagnosis (years)	Diagnosis	Type of HSCT	Age at HSCT (years)	CED g/m2	Radiotherapy		GnRha (triptorelin) treatment	Pharmacological puberty induction	Menarche (years)	Age at last follow-up (years)	Ovarian function at last follow-up
‡1	3.8	Common B-cell ALL	ALLO	10.2	4,2	YES	TBI (12 Gy)	NO	NO	13,0	24.7	normal ovarian function
2	16.0	High-risk BCP-ALL	ALLO	16.6	20,2	NO		YES	NO	13,0	23.6	normal ovarian function
3	17.3	HD	AUTO	18.3	18.7	NO		YES	NO	13.0	21.9	normal ovarian function
4	15.1	CML	ALLO	16.5	3.6	NO		YES	NO	13.0	18.8	normal ovarian function
5	4.9	Common B-cell ALL	ALLO	5.4	5	YES	TBI (12 Gy)	NO	NO	12.0	21.1	normal ovarian function
6	5.6	High-risk T-cell ALL	ALLO	6.3	2.52	NO		NO	NO	12.6	15.4	normal ovarian function
7	15.1	AML M5	ALLO	16.2	42.9	NO		YES	NO	12.0	20.8	normal ovarian function
8	16.6	Common B-cell ALL	ALLO	17.2	6.8	NO		YES	NO	14.0	18.8	normal ovarian function
9	10.8	High-risk ALL	ALLO	11.5	11.8	NO		NO	NO	14.2	14.3	normal ovarian function
‡10	7.6	B-cell ALL	ALLO	11.0	3.8	NO		NO	NO	13.7	14.7	normal ovarian function
11	8.9	AML with granulocytic sarcoma	ALLO	9.3	41.6	NO		NO	NO	11.9	14.3	normal ovarian function
12	13.4	HD	AUTO	14.3	12.5	NO		YES	NO	13.0	21.5	normal ovarian function
13	13.9	# MDS/ AML	ALLO	13.9	37	NO		YES	NO	11.1	21.4	normal ovarian function
‡14	4.3	AML M5	ALLO	9.6	6.3	NO		NO	NO	11.9	14.7	normal ovarian function
15	14.0	AML M4	AUTO	15.3	5.3	NO		YES	NO	13.0	17.8	normal ovarian function
16	7.6	Common B-cell ALL	ALLO	8.4	8.9	YES	TBI (12 Gy)	NO	YES	13.6	17.2	POI
17	9.7	CML	ALLO	9.8	7.1	NO		NO	YES	14.2	33.0	POI
18	8.2	Refractory T-cell ALL	ALLO	8.5	7.5	YES	TBI (12 Gy)	NO	YES	17.5	17.7	POI
‡19	0.7	High-risk AML M5	ALLO	10.8	34.8	YES	CRT (6 Gy) + masseter (27 Gy)	NO	YES	absent	18.8	POI
‡20	7.3	Common B-cell ALL	ALLO	14.4	6.5	YES	TBI (12 Gy)	YES	NO	14	22.6	POI
21	11.3	MPAL	AUTO	11.8	30.4	NO		NO	YES	absent	16.2	POI
*22	3.0	# AML/ALL	AUTO;ALLO	4.0;8.0	30	NO		NO	YES	22.7	29.7	POI
23	9.3	Common B-cell ALL	ALLO	11.4	6.6	YES	TBI (12 Gy)	NO	YES	16.0	25.9	POI
24	9.9	JMML	ALLO	10.1	17.4	NO		NO	YES	15.3	19.7	POI
*25	5.2	PMF with CALR mutation	ALLO	6.1; 6.8	19.2	NO		NO	YES	absent	14.8	POI
26	10.4	High-risk AML M5	ALLO	10.9	30.3	NO		NO	YES	15.1	17.8	POI
27	2.0	HSTCL	ALLO	2.5	25.7	NO		NO	YES	15.8	19.3	POI
‡28	5.5	Common B-cell ALL	ALLO	9.5	6	YES	TBI (12 Gy)	NO	YES	16.7	20.5	TOI
29	2.0	AML M7	ALLO	3.0	22	NO		NO	NO	absent	14.8	TOI
30	7.3	CML	ALLO	8.0	7.5	NO		NO	YES	15.0	24.8	TOI
‡31	4.8	High-risk ALL	ALLO	10.7	6	YES	TBI (12 Gy)	NO	YES	14.5	17.5	TOI
32	10.3	High-risk ALL	ALLO	11.1	3	YES	TBI (12 Gy)	NO	NO	15.4	26.8	TOI
33	5.7	AML M5	ALLO	5.9	18.3	NO		NO	NO	14.2	15.3	TOI
34	12.3	AML M5	ALLO	12.8	18.5	NO		NO	YES	17.8	27.4	TOI

ALL, acute lymphoblastic leukemia; ALLO, allogeneic; AML, acute myeloid leukemia; AUTO, autologous; BCP, B-cell precursor; CALR, calreticulin; CED, cyclophosphamide equivalent dose; CM, chronic myeloid leukemia; CRT, cranial radiation therapy; Gy, Gray; GnRHa, gonadotropin-releasing hormone agonist; HD, Hodgkin disease; HSCT, hematopoietic stem cell transplantation; HSTCL, hepatosplenic T-cell lymphoma; JMML, juvenile myelomonocytic leukemia; MDS, myelodysplastic syndrome; MPAL, mixed phenotype acute leukemia; PMF, primary myelofibrosis; POI, premature ovarian insufficiency; TBI, total body irradiation; TOI, transient ovarian insufficiency.

^‡^Relapse of the primary malignancy.

^#^Sequential hematological malignancies.

*Patients who underwent two HSCT procedures.

**Table 2:** Continuous variables according to current ovarian function status.

Variables	Total population	normal ovarian function	TOI	POI	p-value
Age at disease onset (years)	8.55 (5.28–12.05) (n=34)	13.40 (6.60–15.10) (n=15)	5.70 (5.15–8.80) (n=7)	7.90 (4.65–9.75) (n=12)	0.0559
Age at HSCT (years)	10.75 (8.43–13.62) (n=34)	13.90 (9.90–16.35) (n=15)	9.50 (6.95–10.90) (n=7)	9.95 (8.30–11.03) (n=12)	**0.0432**
Cyclophosphamide equivalent dose (CED) (g/m2)	12.16 (6.35–24.40) (n=34)	11.77 (5.14–22.70) (n=15)	7.46 (6.00–18.41) (n=7)	18.34 (7.40–30.07) (n=12)	0.2650
Age at menarche (years)	14.00 (13.00–15.25) (n=30)	13.00 (12.00–13.00) (n=15)	15.20 (14.62–16.38) (n=6)	15.30 (14.20–16.00) (n=9)	**<0.001**
Age at follow-up (years)	19.08 (16.42–22.42) (n=34)	18.83 (15.04–21.46) (n=15)	20.50 (16.38–25.79) (n=7)	19.04 (17.54–23.42) (n=12)	0.6105
Median time from HSCT to last follow-up (years)	8.46 (4.98–13.79) (n=34)	4.95 (3.18–7.36) (n=15)	11.75 (10.18–15.13) (n=7)	8.97 (8.12–15.10) (n=12)	**0.0031**
AMH (ng/mL)	0.03 (0.01–0.76) (n=31)	0.86 (0.61–1.28) (n=12)	0.03 (0.02–0.17) (n=7)	0.01 (0.01–0.01) (n=12)	**<0.001**
FSH (IU/L)	8.40 (5.90–22.80) (n=33)	6.30 (4.60–8.83) (n=14)	6.18 (5.95–10.50) (n=7)	35.50 (15.72–65.22) (n=12)	**0.0044**
E2 (pg/ml)	47.80 (20.00–109.00) (n=33)	41.95 (20.27–116.28) (n=14)	51.00 (34.05–88.95) (n=7)	51.60 (12.10–111.72) (n=12)	0.9448
Ovarian volume (cm3)	2.75 (1.34–5.04) (n=23)	3.85 (2.48–5.05) (n=15)	1.50 (1.30–3.43) (n=7)	0.36 (0.33–0.73) (n=3)	**0.0068**
Total AFC	3.00 (1.00–5.75) (n=26)	4.00 (2.50–7.50) (n=15)	2.50 (1.25–3.75) (n=6)	0.00 (0.00–0.00) (n=5)	**0.0101**
Uterine volume (cm3)	23.84 (13.82–31.09) (n=34)	29.32 (25.66–53.57) (n=15)	15.48 (14.11–22.97) (n=7)	13.76 (9.76–18.93) (n=12)	**<0.001**
BMD (g/cm2) (L1-L4)	1.11 (0.95–1.22) (n=31)	1.18 (1.15–1.33) (n=13)	1.07 (0.97–1.11) (n=7)	0.96 (0.89–1.12) (n=11)	**0.0187**
Z-score (L1-L4)	-0.10 (-1.70–1.00) (n=31)	1.00 (0.10–1.10) (n=13)	-0.70 (-1.10–-0.25) (n=7)	-2.10 (-2.25–-0.89) (n=11)	**0.0020**

Values are presented as median (Q1–Q3), with the number of non-missing observations shown in parentheses. The Total population column summarizes all patients with available data for a given variable. P-values were calculated using the Kruskal-Wallis test. H, Kruskal-Wallis test statistic; df, degrees of freedom; AMH, anti-Mullerian hormone; BMD, bone mineral density; FSH, follicle-stimulating hormone; E2, estradiol; AFC, antral follicle count; BMD, bone mineral density; CED, cyclophosphamide equivalent dose. Bold valus are p < 0.05.

**Table 3:** Categorical variables according to current ovarian function status.

Variables	Category	Total population	Normal ovarian function	TOI	POI	p-value
Age at transplantation category	Premenarchal	25 (73.5%)	7 (46.7%)	7 (100.0%)	11 (91.7%)	**0.0066**
	Postmenarchal	9 (26.5%)	8 (53.3%)		1 (8.3%)	
Radiotherapy	No	24 (70.6%)	13 (86.7%)	4 (57.1%)	7 (58.3%)	0.1905
	Yes	10 (29.4%)	2 (13.3%)	3 (42.9%)	5 (41.7%)	
GnRHa (Triptorelin)	No	25 (73.5%)	7 (46.7%)	7 (100.0%)	11 (91.7%)	**0.0066**
	Yes	9 (26.5%)	8 (53.3%)		1 (8.3%)	
Puberty induction	No	19 (55.9%)	15 (100.0%)	3 (42.9%)	1 (8.3%)	**<0.001**
	Yes	15 (44.1%)		4 (57.1%)	11 (91.7%)	
Menarche	No	4 (11.8%)		1 (14.3%)	3 (25.0%)	0.1243
	Yes	30 (88.2%)	15 (100.0%)	6 (85.7%)	9 (75.0%)	

Values are presented as n (%). Percentages in the Total population column are calculated using all patients with available data for a given categorical variable. P-values were calculated using Fisher's exact test. GnRHa, Gonadotropin-releasing hormone agonist; POI, premature ovarian insufficiency; TOI, transient ovarian insufficiency. Bold valus are p < 0.05.

The median age at oncological diagnosis was 8.55 years (5.28–12.05), and HSCT was performed at a median age of 10.75 years (8.43–13.62). The median age at follow-up was 19.08 years (16.42–22.42). The median time from HSCT to the last follow-up was 8.46 years (4.98–13.79). Seven patients underwent HSCT following relapse of the primary malignancy, and two patients underwent two HSCT procedures (**Table 1**).

At the last follow-up, patients were classified by current ovarian function status as having normal ovarian function (n = 15, 44.1%), TOI (n = 7, 20.6%), or persistent POI (n = 12, 35.3%). Reclassification from POI to TOI was due to the appearance of follicles in ovarian ultrasound, rapid progression of pubertal development, or the onset of menstruation that was discordant with the prescribed dose of estrogen replacement therapy, along with a rise in E2 levels and a drop in FSH (which at the diagnosis of POI ranged from 36 to 119 IU/L). Diagnosis of TOI resulted in discontinuation of HRT. Among patients with POI receiving HRT without treatment interruption before hormonal assessment, 3 patients used oral estrogen therapy, and 9 received transdermal estrogen preparations, with estimated E2 doses ranging from 25 to 150 μg/day (transdermal).

Patients with normal ovarian function underwent HSCT at an older age than those with TOI or POI (p = 0.0432). Median age at transplantation was 13.90 years (9.90–16.35) in patients with normal ovarian function, 9.50 years (6.95–10.90) in those with TOI, and 9.95 years (8.30–11.03) in those with POI. No significant differences were observed regarding age at disease onset (p = 0.0559), CED (p = 0.2650), or age at follow-up (p = 0.6105). Median CED values were 11.77 g/m² (5.14–22.70), 7.46 g/m² (6.00–18.41), and 18.34 g/m² (7.40–30.07), respectively.

Premenarchal status at HSCT was more common among patients with impaired ovarian function (p = 0.0066). All patients with TOI and 91.7% of patients with POI were premenarchal at transplantation, compared with 46.7% of patients with normal ovarian function.

Ten patients received radiotherapy: 9 underwent TBI (12Gy), and one received cranial (6 Gy) and masseter-region (27 Gy) irradiation. Among the 9 patients exposed to TBI, 7 initially developed POI; however, 3 were reclassified as having TOI at the last follow-up. Although radiotherapy exposure was more common in patients with TOI and POI, no significant association between radiotherapy and ovarian outcome was observed (p = 0.1905).

Puberty induction was strongly associated with impaired ovarian function (p < 0.001). Median age at puberty induction was 13.6 years (13.0–14.75; n = 15). Menarche occurred later in patients with TOI and POI than in those with normal ovarian function (p < 0.001). Median age at menarche was 13.00 years (12.00–13.00) in patients with normal ovarian function, 15.20 years (14.62–16.38) in patients with TOI, and 15.30 years (14.20–16.00) in patients with POI.

AMH concentrations were highest in patients with normal ovarian function and lowest in patients with persistent POI (p < 0.001). Median AMH levels were 0.86 ng/mL (0.61–1.28) in patients with normal ovarian function, 0.03 ng/mL (0.02–0.17) in those with TOI, and 0.01 ng/mL (0.01–0.01) in patients with persistent POI. FSH concentrations were elevated in patients with POI (p = 0.0044), with median values of 6.30 IU/L (4.60–8.83) in patients with normal ovarian function, 6.18 IU/L (5.95–10.50) in those with TOI, and 35.50 IU/L (15.72–65.22) in those with POI. E2 concentrations remained comparable between groups (p = 0.9448). Median E2 levels were 41.95 pg/mL (20.27–116.28) in patients with normal ovarian function, 51.00 pg/mL (34.05–88.95) in those with TOI, and 51.60 pg/mL (12.10–111.72) in patients with POI (**Figure 2**).

**Figure 2:**
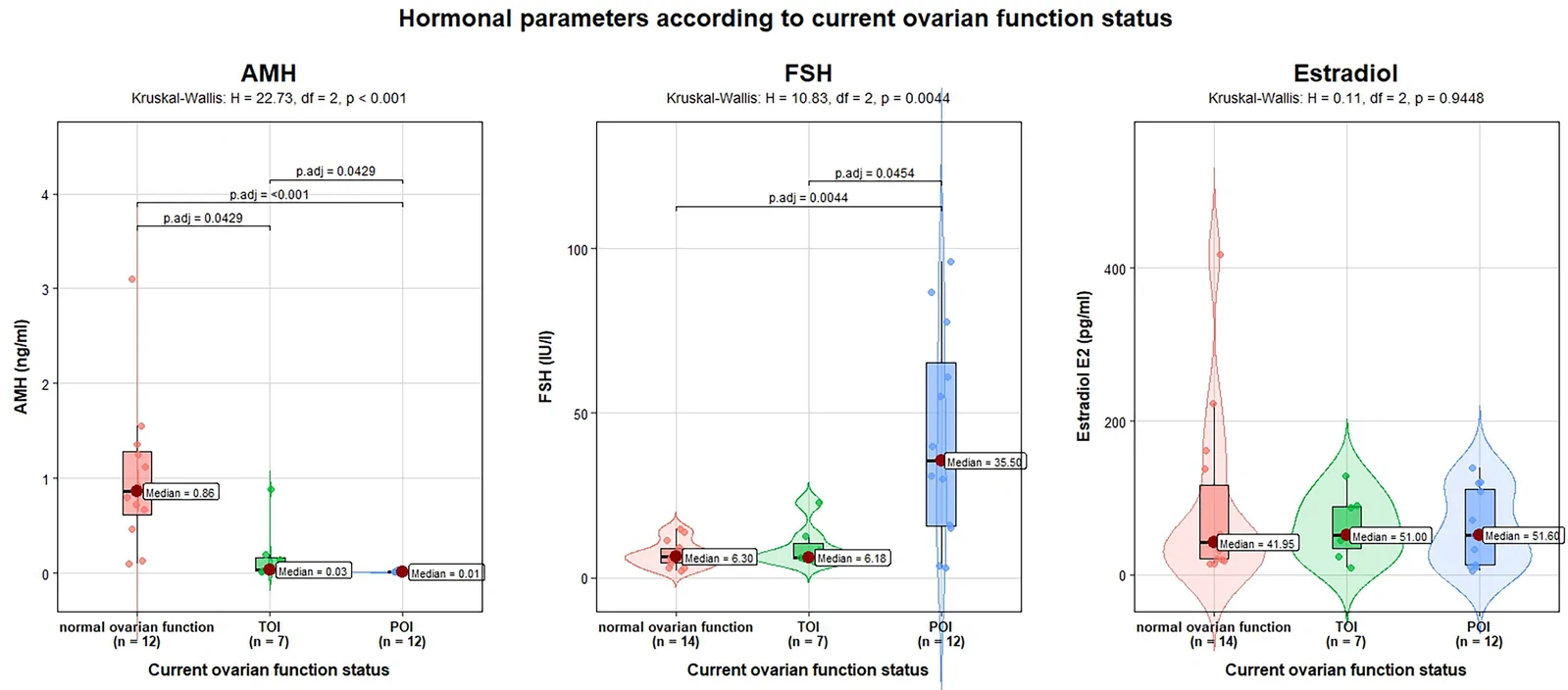
Hormonal parameters according to current ovarian function status.

GnRHa treatment was associated with more favorable ovarian outcomes (p = 0.0066). Per institutional practice, GnRHa was administered only to postmenarchal patients during oncological treatment to induce therapeutic amenorrhea and prevent heavy menstrual bleeding (triptorelin 3.75 mg every 4 weeks or 11.25 mg every 12 weeks). All 9 postmenarchal patients at the time of cancer treatment received GnRHa. Of these, 8 had normal ovarian function at the last follow-up and one was classified as having POI. Among patients with normal ovarian function, those treated with GnRHa had the highest AMH concentrations. Overall, AMH levels were significantly higher in patients treated with GnRHa than in those who did not receive treatment (median 0.92 vs. 0.02 ng/mL, p = 0.0032) (**Figure 3**).

**Figure 3:**
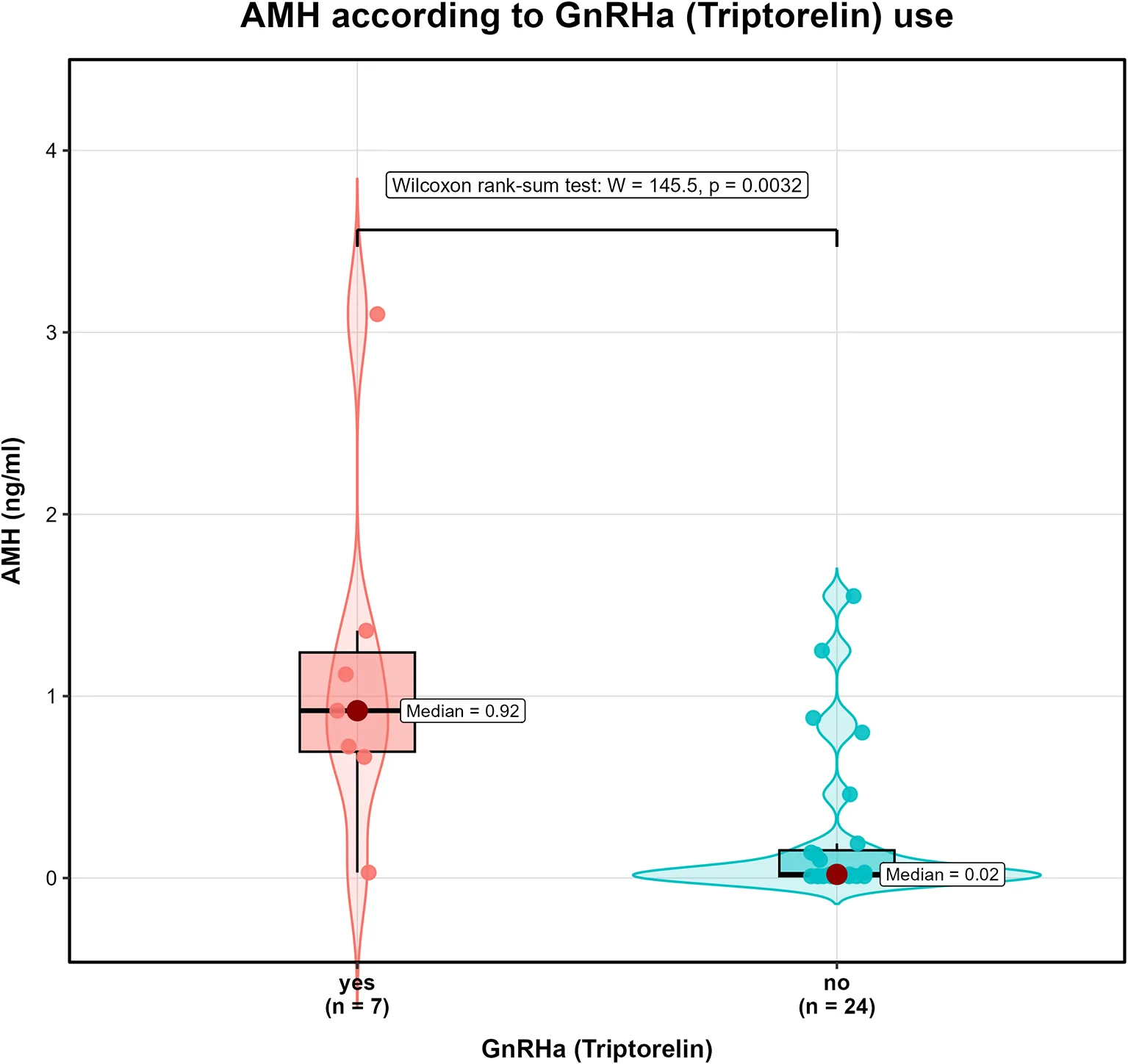
AMH according to GnRHa (Triptorelin use).

The model including CED, radiotherapy, and menarchal status at HSCT was significantly associated with ovarian insufficiency (likelihood-ratio χ²(3) = 12.95, p = 0.0048). Model discrimination was good, with an AUC of 0.821 (95% CI 0.668-0.974) (**Figure 4**). Postmenarchal status at HSCT was independently associated with substantially lower odds of ovarian insufficiency compared with premenarchal status (adjusted OR 0.056, 95% CI 0.005-0.566; p = 0.0147). Neither CED (adjusted OR 1.03 per 1 g/m², 95% CI 0.95-1.12; p = 0.471) nor radiotherapy (adjusted OR 4.87, 95% CI 0.53-45.16; p = 0.163) was independently significant in this model (**Table 4**).

**Figure 4:**
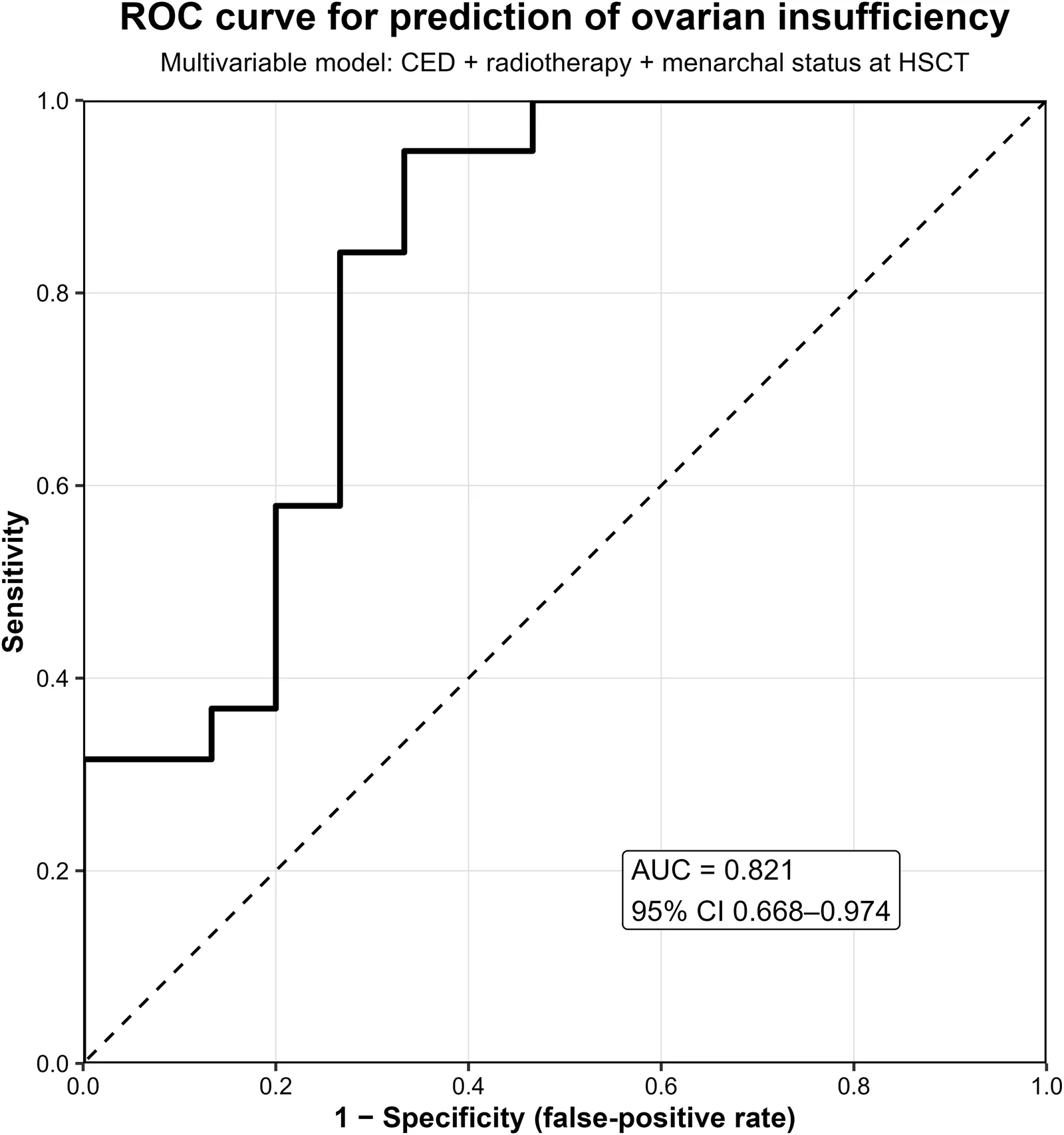
ROC curve for prediction of ovarian insufficiencyMultivariable model: CED + radiotherapy + menarchal status at HSCT.

**Table 4:** Multivariable logistic regression for ovarian insufficiency using menarchal status at HSCT.

Predictor	β	SE	OR	95% CI	p value
CED, per 1 g/m²	0.030	0.042	1.03	0.95-1.12	0.471
Radiotherapy, yes vs no	1.584	1.136	4.87	0.53-45.16	0.163
Postmenarchal vs premenarchal	-2.889	1.184	0.056	0.005-0.566	0.0147

The dependent variable was ovarian insufficiency, defined as TOI or POI versus normal ovarian function. The model included CED, radiotherapy exposure, and menarchal status at HSCT (postmenarchal versus premenarchal). Overall model fit: likelihood-ratio χ²(3) = 12.95, p = 0.0048; AIC = 41.72; McFadden pseudo-R² = 0.277; AUC = 0.821 (95% CI 0.668-0.974). ORs are adjusted odds ratios. CED was modeled per 1 g/m².

Uterine volume was lower in patients with impaired ovarian function (p < 0.001). The median uterine volume was 29.32 cm³ (25.66–53.57) in patients with normal ovarian function, 15.48 cm³ (14.11–22.97) in patients with TOI, and 13.76 cm³ (9.76–18.93) in patients with POI. Patients who received radiotherapy had lower uterine volumes than those who did not (11.79 vs. 26.30 cm³, p < 0.001). Similarly, uterine volume was lower in patients who required puberty induction than in those with spontaneous pubertal development (14.69 vs. 26.32 cm³, p = 0.0020) (**Figure 5**).

**Figure 5:**
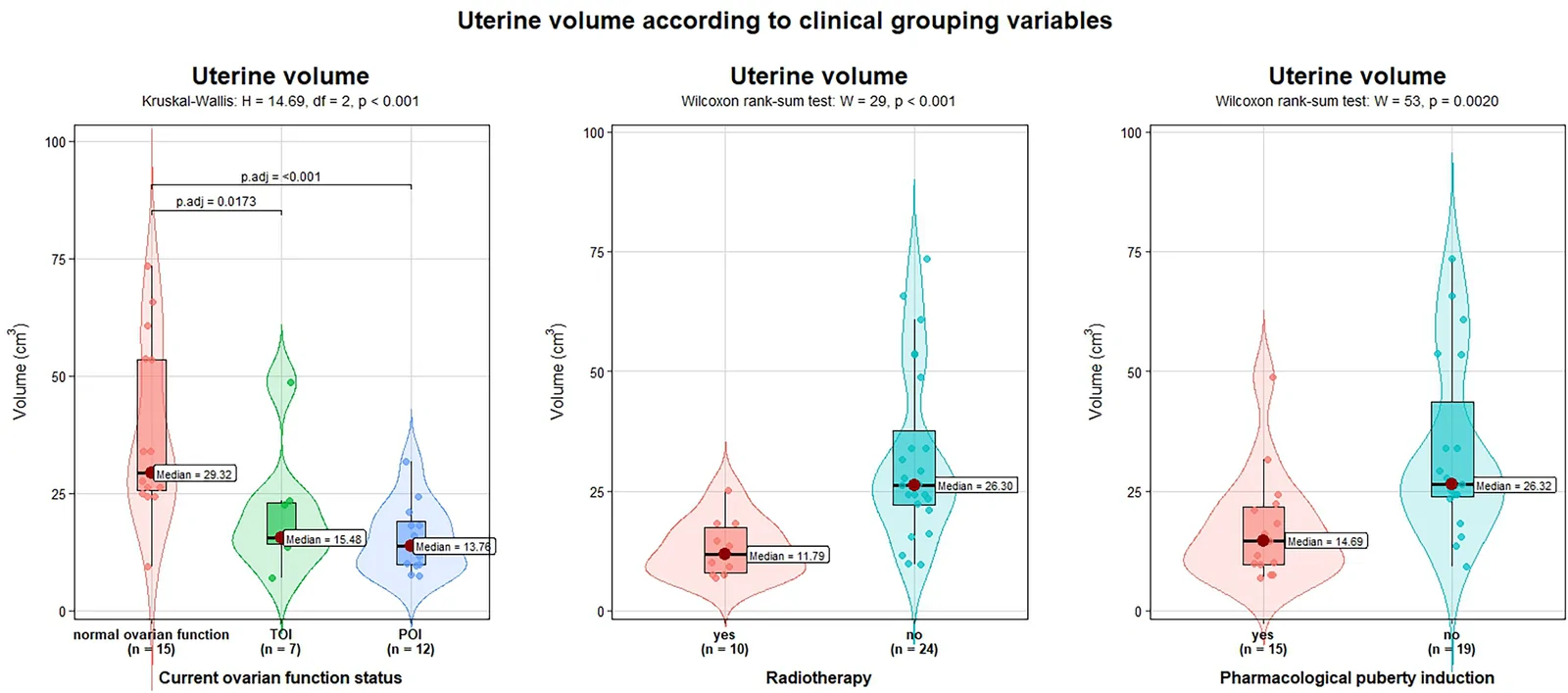
Uterine volume according to clinical grouping variables.

Ultrasound markers of ovarian reserve differed significantly according to ovarian function status. Mean ovarian volume (defined as the mean volume of the left and right ovary) decreased progressively from patients with normal ovarian function to those with TOI and POI (p = 0.0068), with significantly lower values observed in patients with POI than in those with normal ovarian function (p = 0.0077). Similarly, total AFC was highest in patients with normal ovarian function and lowest in those with POI (p = 0.0101) (**Figure 6**).

**Figure 6:**
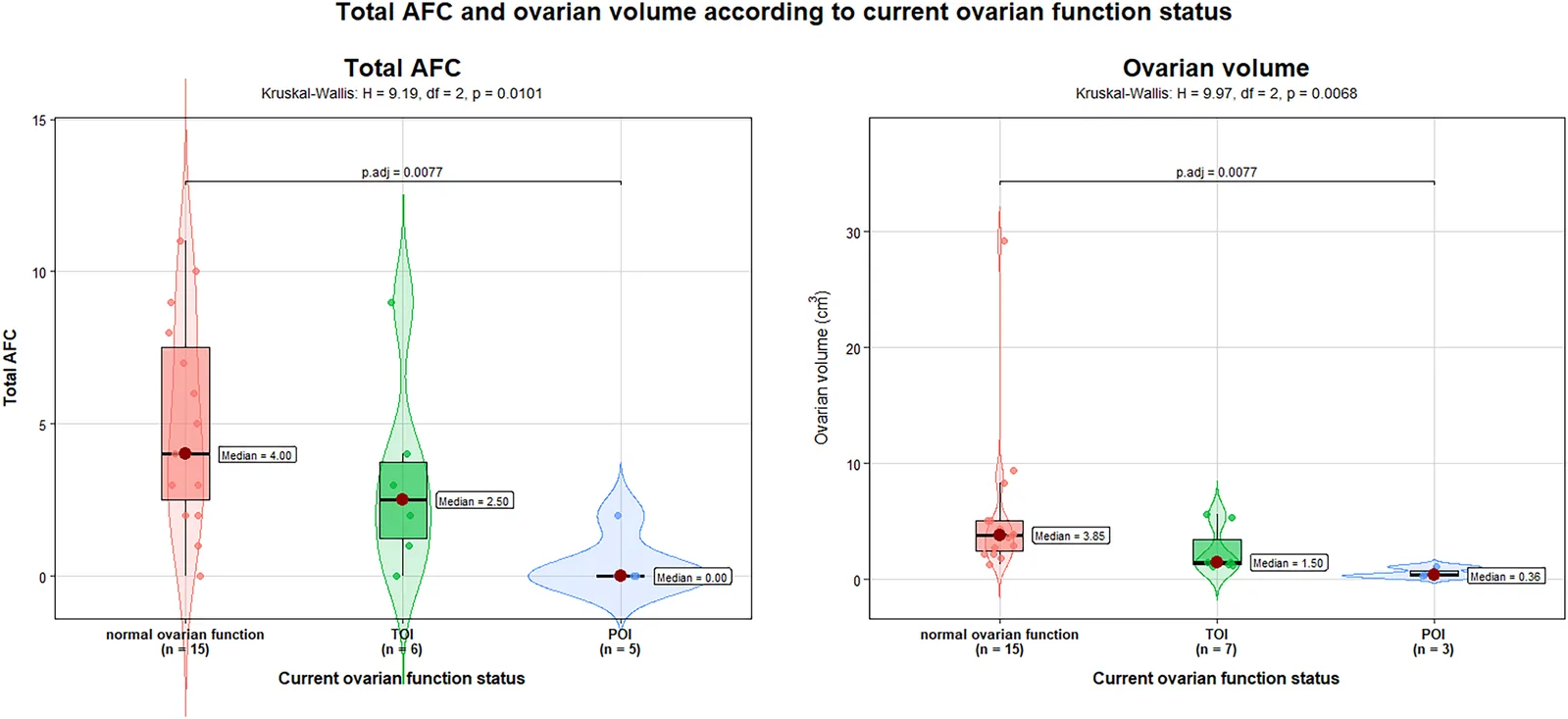
Total AFC and ovarian volume according to current ovarian function status.

Mean ovarian volume showed a strong positive correlation with total AFC (Spearman’s ρ = 0.894, p < 0.001; linear regression p < 0.001) (**Figure 7**). In addition, patients exposed to radiotherapy had significantly lower mean ovarian volume than those without radiotherapy (median 1.32 vs. 3.95 cm³, p = 0.0020) (**Figure 8**).

**Figure 7:**
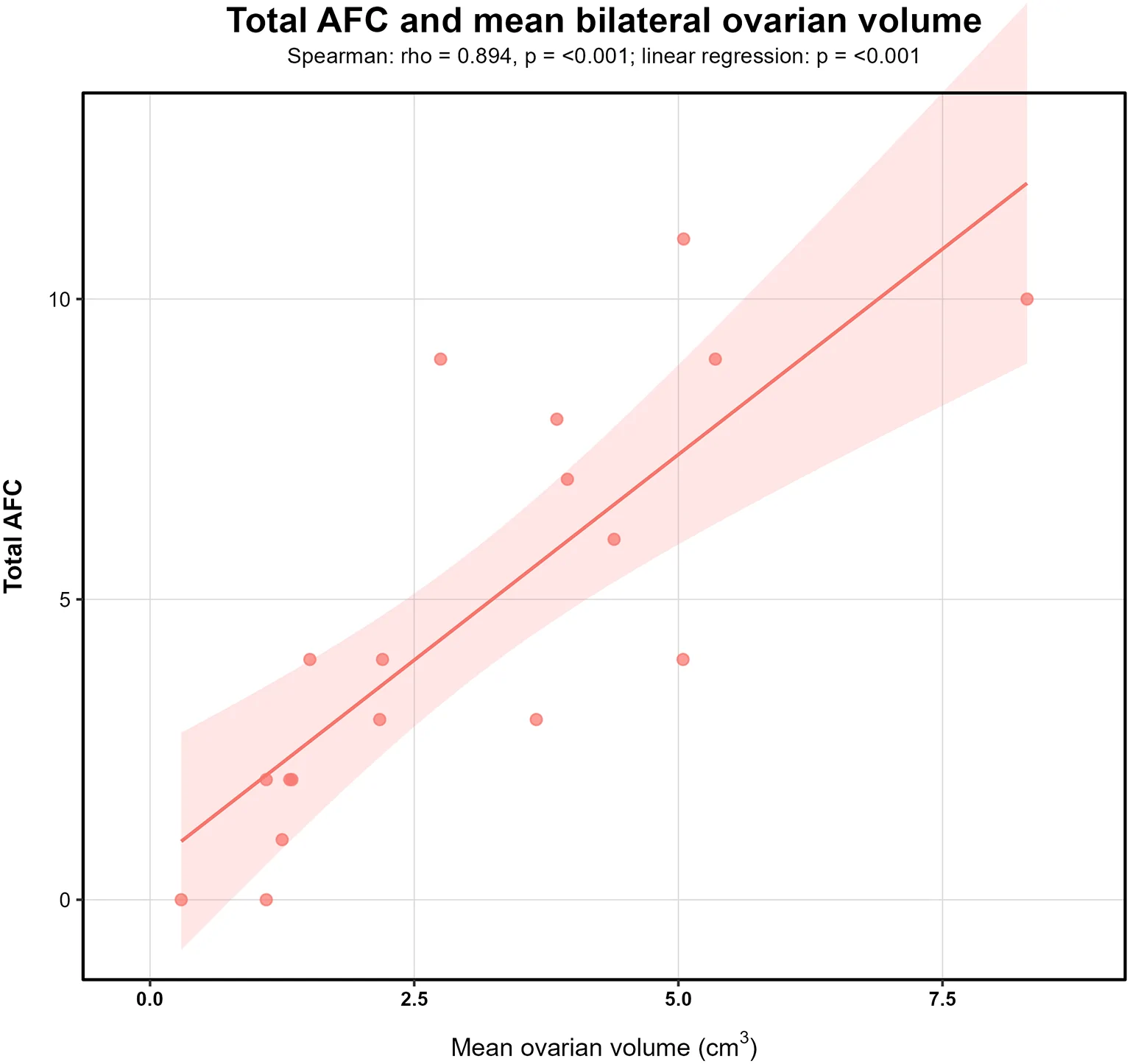
Total AFC and mean bilateral ovarian volume.

**Figure 8:**
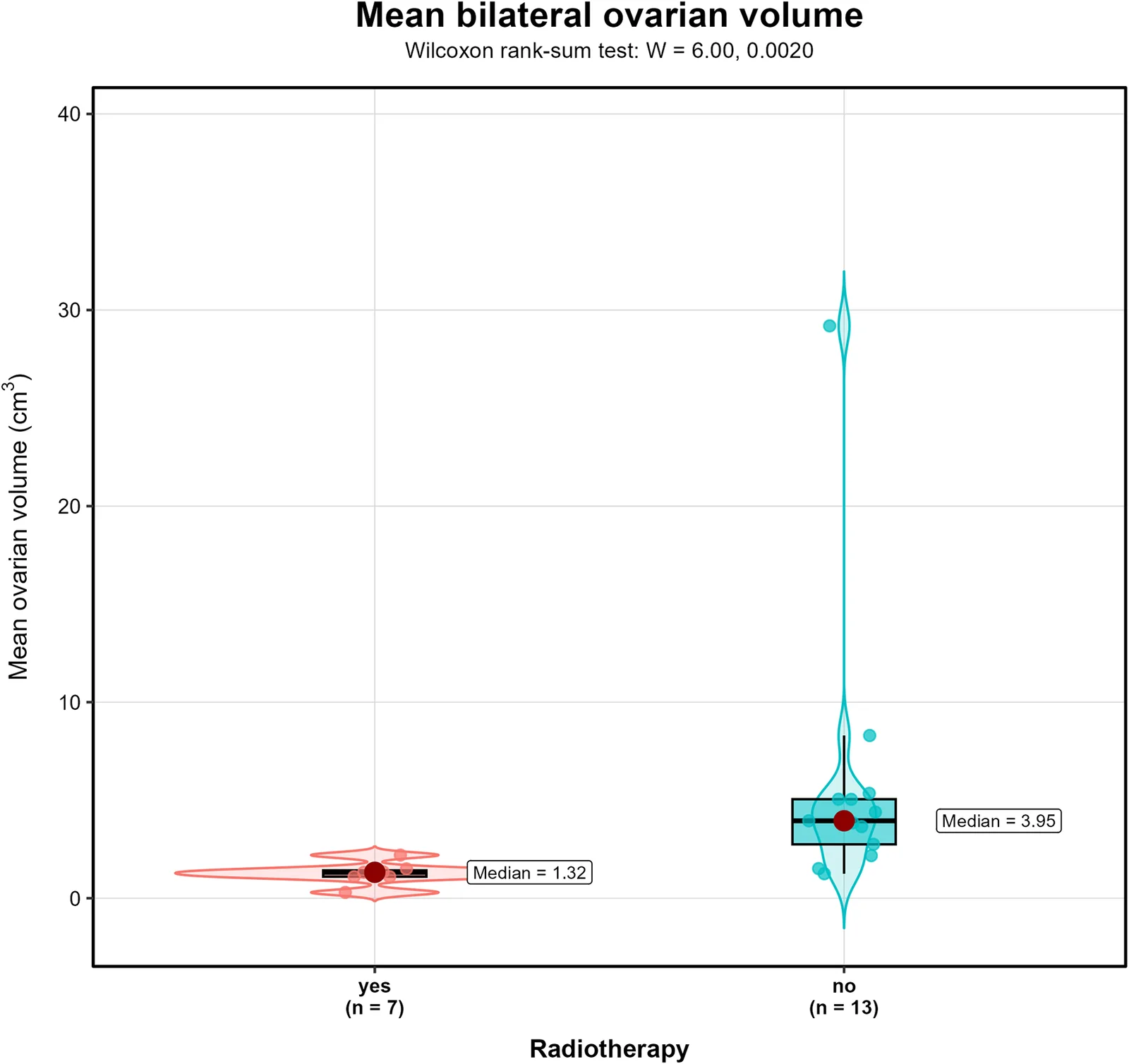
Mean bilateral ovarian volume.

Among patients with TOI, the median time from completion of cancer treatment to TOI diagnosis was 11.8 years. Paired AMH measurements were available for 6 patients. The median AMH increased from 0.01 ng/mL at the initial assessment to 0.08 ng/mL at the last follow-up; however, individual trajectories were heterogeneous, and the paired change was not statistically significant (p = 0.4185) (**Figure 9**). Considering all available AMH measurements, the overall median AMH was higher at the last assessment than at baseline (0.03 vs. 0.01 ng/mL), although this difference did not reach statistical significance (p = 0.0709). Total AFC remained stable over time, with median values of 3.00 and 2.50, respectively (p = 1.0000) (**Figure 10**). Nevertheless, individual patients showed partial recovery of ovarian reserve markers over time.

**Figure 9:**
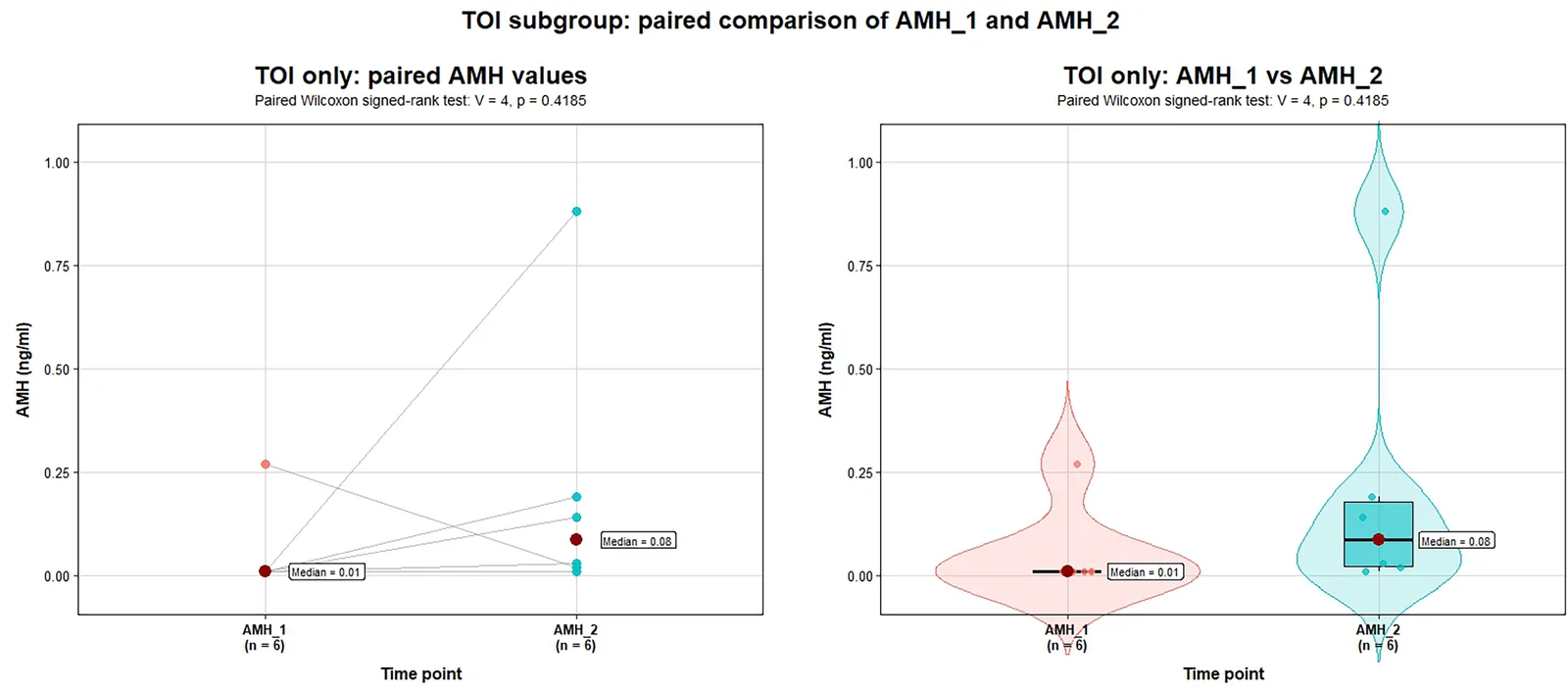
TOI subgroup: paired comparison of AMH_1 and AMH_2.

**Figure 10:**
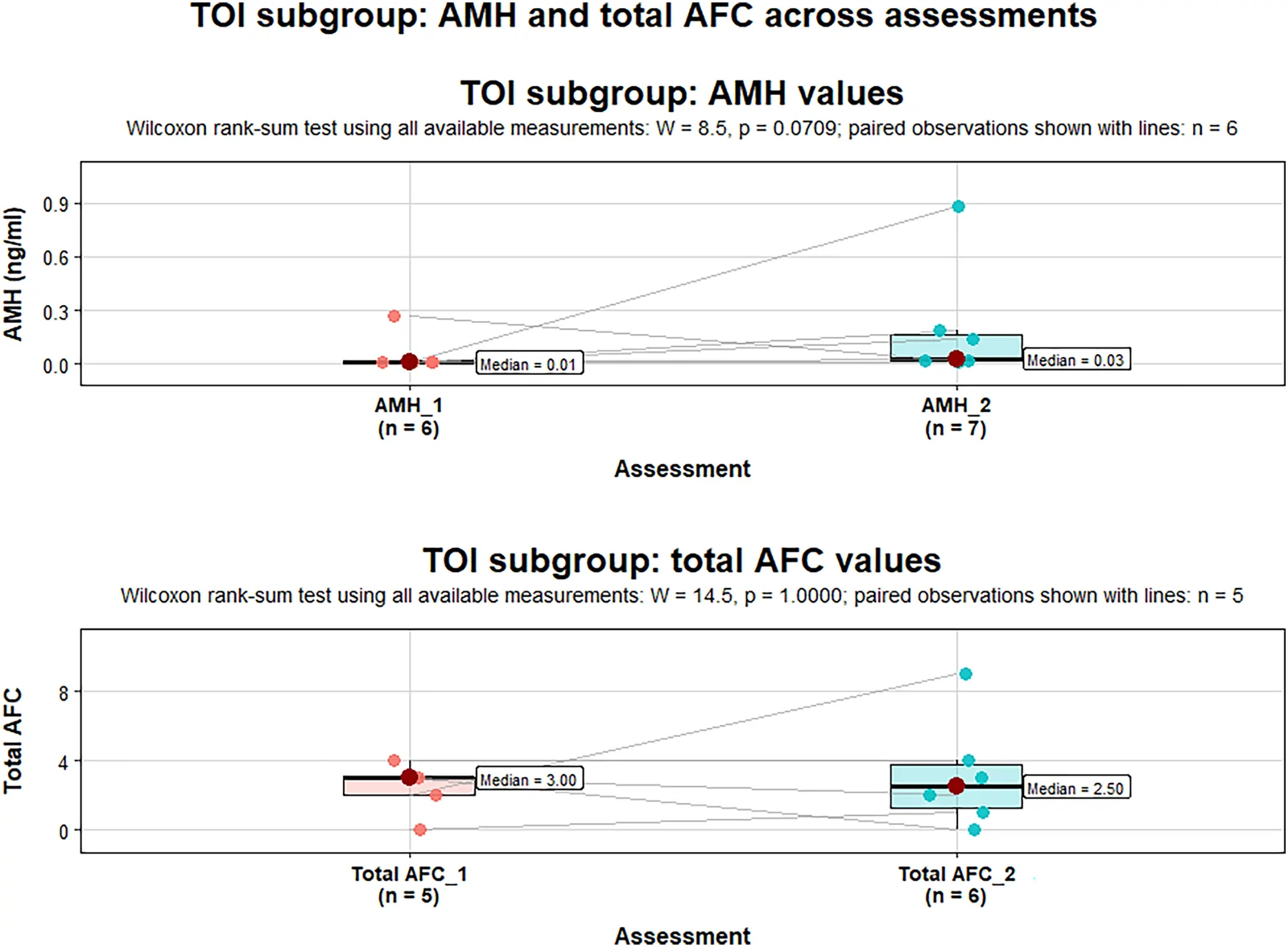
TOI subgroup: AMH and total AFC across assessments.

Lumbar spine (L1–L4) Z-scores were lowest in patients with persistent POI (p = 0.0020). Median Z-scores were 1.00 (0.10–1.10) in patients with normal ovarian function, −0.70 (−1.10 to −0.25) in those with TOI, and −2.10 (−2.25 to −0.89) in patients with POI. Bone mineral density values also decreased progressively across ovarian function groups (p = 0.0187). Median BMD values were 1.18 g/cm² (1.15–1.33) in patients with normal ovarian function, 1.07 g/cm² (0.97–1.11) in those with TOI, and 0.96 g/cm² (0.89–1.12) in patients with POI (**Figure 11**).

**Figure 11:**
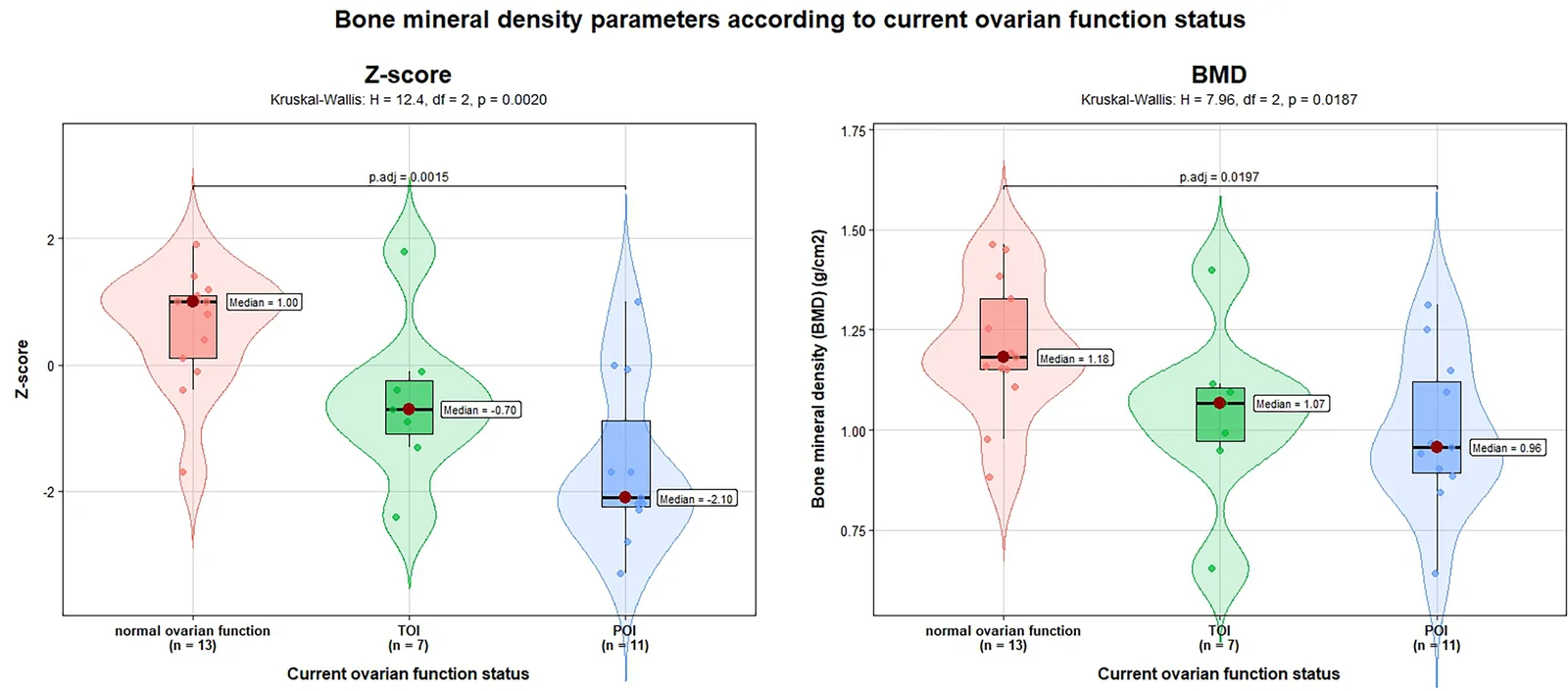
Bone mineral density parameters according to current ovarian function status.

Two patients achieved spontaneous pregnancies during follow-up. Patient no. 17, who remained classified as having POI throughout follow-up because of persistently elevated FSH concentrations, experienced an early miscarriage and is currently awaiting *in vitro* fertilization (IVF) with donor oocytes. Patient no. 32, who was reclassified from POI to TOI following biochemical recovery of ovarian function, delivered at 36 weeks of gestation; the neonate’s weight was appropriate for gestational age. The pregnancy was complicated by gestational diabetes requiring insulin therapy.

## Discussion

Ovarian dysfunction was highly prevalent in our cohort, with 55.9% of patients initially diagnosed with POI. However, ovarian insufficiency was not static, as seven patients later demonstrated partial recovery of ovarian function and were subsequently reclassified as having TOI. These findings support the concept that ovarian function after gonadotoxic treatment may fluctuate over time and should not always be considered permanently absent following an initial diagnosis of POI. The extended follow-up period represents one of the major strengths of our study. The median time from completion of cancer treatment to TOI diagnosis was 11.8 years, emphasizing the importance of long-term surveillance and comprehensive assessment in this population. The delayed recovery suggests that residual follicular activity may persist for years after gonadotoxic treatment and become clinically apparent only during extended follow-up.

Younger age at treatment is generally considered protective for ovarian function, and previous studies have identified HSCT performed after the age of 10 years as an independent risk factor for premature ovarian insufficiency (POI) (17). Consistent with these findings, a French study by Chabut et al. involving 178 women who underwent HSCT for leukemia before puberty demonstrated that transplantation before the age of 4.8 years was associated with better preservation of ovarian function, with nearly 50% of patients remaining free of POI at last follow-up, whereas transplantation after the age of 10.9 years was associated with a substantially higher risk of ovarian failure, with more than 85% of patients requiring hormone replacement therapy (HRT) (18). Similar results were reported by Lee et al. in a retrospective Australian single-center study involving 54 females who received HSCT for malignant disease between 1985 and 2011. Within this subgroup, females who retained ovarian function were significantly younger at HSCT than those who developed POI (median age 4.9 vs. 10.1 years), while pubertal status at transplantation was not associated with ovarian function (19).

However, our findings do not fully support this assumption. Patients with normal ovarian function were significantly older at HSCT than those with TOI and POI, whereas premenarchal status at transplantation was more common in the subgroup with impaired ovarian outcome. The median age at HSCT among premenarchal patients in our cohort was 9.7 years, suggesting that some may already have been in the peripubertal stage at the time of transplantation despite not having achieved menarche. As Tanner stage data were not collected during HSCT, pubertal maturation beyond menarche status could not be assessed. Despite this limitation, a substantial proportion of patients who were premenarchal at HSCT developed POI, confirming that absence of menarche does not preclude substantial ovarian vulnerability to gonadotoxic therapy. Among our patients who were premenarchal at HSCT, POI was observed in 44%, TOI in 28%, and normal ovarian function in only 28%. In contrast, among those who had already achieved menarche before HSCT, normal ovarian function was preserved in 88% of cases, with POI observed in only 12%. Thus, the expected protective association of younger age and premenarchal status was not observed in our cohort.

Importantly, in our cohort, GnRHa was administered exclusively to postmenarchal patients. All 9 postmenarchal patients received GnRHa, and 8 maintained normal ovarian function at the last follow-up. This complete overlap between menarchal status and GnRHa exposure provides an important context for interpreting the unexpected association between more advanced developmental status at HSCT and favorable ovarian outcomes. In multivariable analysis accounting simultaneously for CED, radiotherapy exposure, and menarchal status at HSCT, postmenarchal status remained associated with markedly lower odds of subsequent ovarian insufficiency. However, because all postmenarchal patients received GnRHa, the effects of menarchal status and GnRHa exposure cannot be disentangled, and the observed association cannot be attributed to menarchal status itself. The more favorable outcomes observed among postmenarchal patients may therefore reflect, at least in part, the selective administration of GnRHa. Consistent with this possibility, GnRHa-treated patients had significantly higher AMH concentrations than those who did not receive GnRHa.

Data on the impact of GnRHa co-treatment on long-term ovarian function after childhood cancer therapy remain scarce. Existing reports are mainly based on small retrospective cohorts of adolescents, some of which have suggested improved menstrual recovery and higher post-treatment AMH levels following GnRHa administration (20). Nevertheless, findings remain inconsistent, particularly among patients exposed to highly gonadotoxic treatments. In a retrospective study of postmenarchal adolescents undergoing HSCT after cancer treatment, POI was diagnosed in 44% of patients (4/9) despite GnRHa co-treatment, suggesting that any potential protective effect may be limited in this high-risk population (21). Although our findings are compatible with a potential benefit of GnRHa, they cannot establish an independent protective effect. Further studies are required, and GnRHa should not currently be considered an established method of fertility preservation (4). The ongoing ProFertil study in Sweden is a phase III randomized trial designed to address this question (22).

CED was not independently associated with ovarian insufficiency in the multivariable model. Although CED exceeding 8 g/m² in postmenarchal and 12 g/m² in premenarchal patients is considered highly gonadotoxic (13), 8 of 15 patients with normal ovarian function received doses above these thresholds without developing POI. This variability suggests that cumulative alkylating-agent exposure alone may not fully explain individual ovarian outcomes after HSCT.

TBI is considered one of the most gonadotoxic components of conditioning regimens prior to HSCT, with doses of 10–12 Gy resulting in an approximately 80% risk of POI (23). Molinari et al. observed ovarian injury in almost 100% of patients exposed to TBI before allo-HSCT (9). More recently, Su et al. diagnosed POI in 90% of patients who received TBI before transplantation, compared with 33% of those who did not (24). Consistent with these findings, all 24 female patients exposed to TBI in the study by Lee et al. developed POI (20). In our cohort, radiotherapy exposure was associated with higher odds of impaired ovarian function (POI/TOI), although the association was not statistically significant in the unadjusted analysis and remained non-significant after adjustment for CED and menarchal status at HSCT. However, given the limited sample size, this lack of statistical significance should not be interpreted as evidence of the absence of a gonadotoxic effect. Remarkably, 43% of patients exposed to TBI who were initially diagnosed with POI subsequently showed recovery of ovarian activity and were reclassified as having TOI. These findings further support the concept that ovarian function after gonadotoxic treatment may remain dynamic rather than irreversible.

The likelihood of spontaneous conception in patients with POI is less than 5%, and oocyte donation is generally regarded as the first-line option for achieving pregnancy (4). However, Chabut et al. demonstrated that natural conception remains possible after childhood HSCT, reporting at least one spontaneous pregnancy in 22 of 178 women (12%) transplanted before puberty (18). Lee et al. reported spontaneous pregnancies resulting in eight live births among 6 of 37 sexually active female survivors of childhood allogeneic HSCT (16.2%), including three women previously exposed to TBI (25). In our cohort, two patients attempted pregnancy, and both conceived spontaneously. Patient no. 17 conceived despite continuously elevated FSH concentrations and remained classified as having POI throughout follow-up; the pregnancy ended in an early miscarriage. In contrast, patient no. 32, who had been reclassified from POI to TOI following biochemical recovery of ovarian function, achieved a spontaneous pregnancy despite persistently low AMH levels (0.01 ng/mL). The pregnancy was complicated by gestational diabetes, and she delivered a live infant at 36 weeks of gestation (late preterm). These observations suggest that conception may remain possible even after prolonged ovarian insufficiency and in the presence of markedly diminished ovarian reserve. However, reproductive outcomes may still be adversely affected by prior cancer treatment. This is supported by a recent systematic review and meta-analysis demonstrating an increased risk of obstetric complications among cancer survivors, including a 37% higher risk of preeclampsia and a 29% higher risk of gestational diabetes, particularly following abdominal or pelvic radiotherapy (26). Furthermore, radiation exposure has been associated with preterm delivery and fetal growth restriction (5, 27), indicating that reproductive outcomes may remain compromised even in patients who retain partial ovarian function; reproductive outcomes may also be compromised by uterine factors.

In addition to its gonadotoxic effects, radiotherapy may adversely affect uterine development. Radiation doses ≥12 Gy have been associated with impaired uterine growth, reduced uterine volume, and an increased risk of severe uterine hypoplasia, particularly when exposure occurs during the prepubertal period (5, 28, 29). Consistent with these findings, patients in our cohort who received radiotherapy had significantly lower uterine volumes than non-irradiated patients. Furthermore, patients who required pubertal induction reached menarche later than those who underwent spontaneous pubertal development. Even after HRT, uterine volume remained significantly lower than in patients with spontaneous puberty, suggesting impaired uterine development following gonadotoxic therapy and possibly reduced responsiveness to exogenous E2 compared with endogenous hormonal stimulation. The median age at puberty induction was 13.6 years, which is later than the physiological onset of puberty, and may further reduce cumulative estrogen exposure during adolescence.

AMH and FSH concentrations clearly reflected ovarian function in this cohort. AMH levels were highest in patients with normal ovarian function and lowest in those with persistent POI, whereas FSH concentrations were markedly elevated in POI. In contrast, E2 concentrations at the last follow-up did not differ between the groups. In our study, HRT was not discontinued before hormonal assessment in patients previously diagnosed with POI. As patients received either transdermal or oral E2, the lack of differences in E2 concentrations likely reflects appropriate individualization of HRT. Despite ongoing HRT and variability in estrogen administration routes, FSH concentrations remained significantly different between groups, suggesting persistent differences in ovarian endocrine activity. These findings indicate that FSH may still provide clinically useful information regarding ovarian function even in patients receiving estrogen replacement therapy.

AFC findings further supported the biological differences between ovarian function groups. Total AFC was significantly lower in patients with TOI and POI than in those with normal ovarian function, with the lowest values (most often undetectable AF) observed in patients with persistent POI. Both total AFC and mean ovarian volume were lower in patients with impaired ovarian function, and a strong positive correlation was observed between these two parameters, further demonstrating the consistency of sonographic markers of ovarian impairment in this cohort. Moreover, patients exposed to radiotherapy had significantly lower ovarian volumes than those who were not irradiated, consistent with the gonadotoxic effects of radiation. Notably, AF remained detectable in several patients with TOI despite persistently very low AMH concentrations, suggesting that ultrasound assessment may detect signs of ovarian activity that are not fully reflected by AMH concentrations. Therefore, AFC, complemented by assessment of ovarian morphology, may provide additional clinically relevant information regarding ovarian reserve and help identify patients with partial ovarian recovery during long-term follow-up.

Previous studies have highlighted that ovarian reserve may be substantially impaired in childhood cancer survivors despite apparent recovery of endocrine function, and that menstrual function alone may not accurately reflect reproductive potential (30, 31). In line with these reports, patients with apparently preserved ovarian function in our cohort exhibited relatively low AMH concentrations (median 0.86 ng/mL) despite regular menstrual cycles, FSH concentrations within the normal range, and detectable AF, suggesting diminished ovarian reserve. These observations highlight the limitations of relying solely on menstrual history or gonadotropin concentrations when assessing gonadal function after HSCT. A comprehensive evaluation should therefore include both AMH and AFC, as ovarian reserve may be substantially reduced despite apparently preserved ovarian function. Besides, such results may justify secondary fertility preservation in AYAs who have not undergone ovarian tissue or oocyte cryopreservation before oncological treatment.

Notably, the clinical consequences of ovarian dysfunction extended beyond reproductive markers. While untreated ovarian insufficiency has been associated with reduced peak bone mass and increased long-term morbidity (32), patients with persistent POI in our cohort exhibited significantly lower lumbar spine (L1–L4) BMD despite ongoing HRT. Although E2 concentrations were comparable across ovarian function groups, reflecting ongoing estrogen supplementation in patients with POI, reduced BMD persisted in this subgroup. This finding suggests that the adverse skeletal consequences of POI may not be fully reversed by HRT. Reduced BMD may reflect prolonged hypoestrogenism before treatment initiation, impaired acquisition of peak bone mass during adolescence, shorter cumulative estrogen exposure, or incomplete restoration of physiological estrogen effects despite HRT. These results emphasize that POI is associated with long-term health consequences extending beyond fertility and reproductive function, underscoring the importance of early diagnosis, timely initiation of HRT, and regular bone health monitoring.

Several limitations of our study should be acknowledged. The relatively small sample size reflects the challenges inherent in this patient population rather than the study design. Moreover, the study was intentionally restricted to female survivors who underwent HSCT for hematological malignancies at a single tertiary center, representing a highly selected and relatively uncommon clinical population. In Poland, comprehensive gynecological care for pediatric cancer survivors remains limited, particularly after transition to adult care, which hampers long-term follow-up. Patient recruitment was further constrained by the General Data Protection Regulation (GDPR), as consent for future contact and long-term follow-up is not routinely obtained during oncological treatment. Consequently, the long interval since HSCT limited our ability to re-contact many eligible survivors. In addition, several eligible survivors declined participation because they were already under regular gynecological care and did not perceive an additional study visit as beneficial. Conversely, survivors experiencing menstrual or reproductive concerns appeared more willing to participate. Therefore, selection bias cannot be excluded, and the prevalence of ovarian dysfunction observed in our cohort may have been overestimated.

## Conclusions

POI is a common long-term complication of childhood HSCT. However, ovarian function remains dynamic, and partial recovery may occur even years after treatment. Although patients with TOI show recovery of ovarian function and residual ovarian activity, they still exhibit reduced ovarian reserve, lower BMD, and impaired ultrasound parameters compared with patients with normal ovarian function, indicating that TOI does not represent complete restoration of ovarian health.

Assessment of ovarian function should extend beyond menstrual history and FSH concentrations, as both may fail to detect diminished ovarian reserve in survivors with apparently preserved ovarian function. Long-term follow-up should therefore include AMH, AFC, and a comprehensive reproductive assessment. AFC may provide additional information on residual ovarian activity and early ovarian recovery.

GnRHa administration may improve long-term ovarian outcomes in postmenarchal female patients undergoing gonadotoxic therapy. Nevertheless, larger prospective, randomized studies are needed to confirm this observation and clarify the role of GnRHa in preserving ovarian function.

## Data Availability

The raw data supporting the conclusions of this article will be made available by the authors, without undue reservation.
